# Role of Creatine Kinase in the Troponin Era: A Systematic Review

**DOI:** 10.5811/westjem.2020.11.47709

**Published:** 2021-10-27

**Authors:** Daniel Beamish, Tetyana Maniuk, Muhammad Mukarram, Venkatesh Thiruganasambandamoorthy

**Affiliations:** * University of Ottawa, Department of Emergency Medicine, Ottawa, Ontario, Canada; † The Ottawa Hospital, Ottawa Hospital Research Institute, Ottawa, Ontario, Canada; ‡ University of Ottawa, School of Epidemiology and Public Health, Ottawa, Ontario, Canada

## Abstract

**Introduction:**

The diagnosis of non-ST-elevated myocardial infarction (NSTEMI) depends on a combination of history, electrocardiogram, and cardiac biomarkers. The most sensitive and specific biomarkers for cardiac injury are the troponin assays. Many hospitals continue to automatically order less sensitive and less specific biomarkers such as creatine kinase (CK) alongside cardiac troponin (cTn) for workup of patients with chest pain. The objective of this systematic review was to identify whether CK testing is useful in the workup of patients with NSTEMI symptoms.

**Methods:**

We undertook a systematic review to ascertain whether CK ordered as part of the workup for NSTEMI was useful in screening patients with cardiac chest pain. The MEDLINE, Embase, and Cochrane databases were searched from January 1995–September 2020. Additional papers were added after consultation with experts. We screened a total of 2,865 papers, of which eight were included in the final analysis. These papers all compared CK and cTn for NSTEMI diagnosis.

**Results:**

In each of the eight papers included in the analysis, cTn showed a greater sensitivity and specificity than CK in the diagnosis of NSTEMI. Furthermore, none of the articles published reliable evidence that CK is useful in NSTEMI diagnosis when troponin was negative.

**Conclusion:**

There is no evidence to continue to use CK as part of the workup of NSTEMI acute coronary syndrome in undifferentiated chest pain patients. We conclude that CK should not be used to screen patients presenting to the emergency department with chest pain.

## INTRODUCTION

Chest pain is a common emergency department (ED) presenting complaint.^1^ The objective of ED evaluation is to rule out acute coronary syndrome (ACS), which comprises ST-elevated myocardial infarction (STEMI), non-ST-elevated myocardial infarction (NSTEMI), and unstable angina. A clinical history and/or electrocardiogram (ECG) is used for diagnosis of STEMI and unstable angina. Non-ST-elevated myocardial infarction constitutes 70% of ACS and is diagnosed using biomarkers.^2^–^4^ The biomarkers used to diagnose NSTEMI have evolved greatly over the last 50 years. They have changed from the relatively non-specific biomarkers such as aspartate aminotransferase, lactate dehydrogenase, myoglobin, and creatine kinase (CK) (and its cardiac isoform CK-MB) to the very sensitive and specific cardiac troponin assays (TnI, TnT).^5^–^6^ Despite the availability and use of sensitive and specific cardiac troponin (cTn) biomarker assays, many physicians continue to order CK for ACS diagnosis as well, notwithstanding recommendations to the contrary.^7^,^8^ The objective of this systematic review was to identify whether CK testing is useful in the workup of patients with NSTEMI symptoms.

## METHODS

### Search Strategy and Study Selection

We conducted a systematic search using the Cochrane Library, Embase (OVID) and Medline (OVID) databases from January 1, 1995–September 2020. We included prospective and retrospective studies that measured CK levels as part of chest pain evaluation and compared it to cTn levels for NSTEMI diagnosis (Appendix A). The diagnosis of NSTEMI was dependent upon the institution and included World Health Organization (WHO) classification (at that time), as well as the diagnosis made by consulting cardiologists or staff physicians. We restricted our review to English-language and human studies. We excluded articles that compared CK to CK-MB to novel biomarkers that are not cTn, studies that used CK to evaluate infarct size in the setting of STEMI rather than NSTEMI diagnosis, and studies that included post-intervention patients (stent insertion or lytic administration). We also excluded studies involving children, special populations (eg, marathon runners), case reports, letters to the editor, and narrative reviews, or if data abstraction was not possible. The grey literature of unpublished abstracts was not searched.

### Data Abstraction

Article titles and abstracts were independently screened by two review authors (DB, TM). Both reviewers independently screened full texts of potentially relevant studies. Disagreements were discussed between the two reviewers, and decisions were reached by consensus and adjudicated by a third reviewer (VT). We reviewed the bibliography of included articles and consulted authors to identify potentially missed studies. If data were missing we contacted authors a minimum of two times, two weeks apart via email. We used Covidence systematic review software (Veritas Health Innovation Ltd, Melbourne, Australia) to track articles in the systematic review. Our outcome was NSTEMI diagnosis. We assessed the diagnostic characteristics of troponin and CK in NSTEMI diagnosis.

### Data Extraction and Quality Assessment

We extracted data for calculation of diagnostic characteristics using 2 × 2 tables. We specifically aimed to identify patients with a final NSTEMI diagnosis who had a negative cTn and elevated CK on initial evaluation. Quality assessment of the included studies was done using the quality assessment of diagnostic accuracy studies tool^17^ (QUADAS-2, developed collaboratively by the Centre for Reviews and Dissemination, University of York, and the Academic Medical Centre at the University of Amsterdam). We adhered to the Preferred Reporting Items for Systematic Reviews and Meta-Analyses statement for systematic reviews.

### Data Synthesis and Analysis

Owing to the small number of total trials with complete extractable data and the heterogeneity, a pooled meta-analysis would not be statistically valid. We therefore opted for a descriptive analysis of the data.

## RESULTS

We identified 2,862 studies by the initial search strategy, and an additional three articles were identified by an author who was contacted for data clarification, leading to a total of 2,865 studies (Appendix B). Of those, 2,664 studies did not meet inclusion criteria, leaving 201 articles for full-text review stage. Of the 201 papers included in full-text review 193 studies were excluded. We identified a total of eight articles that met our inclusion criteria for the review process (Table 1). Three included studies were NSTEMI databases,^9^–^11^ wherein patients with confirmed NSTEMI were analyzed and their biomarkers were studied retrospectively. The remaining five papers consisted of patient groups that either were admitted for suspected ACS or were being evaluated for ACS in the ED.^12^–^15^

All papers that compared CK and cTn found that cTn was more sensitive than CK, regardless of the timing of their measurement (Appendix C). Sensitivity of troponin ranged from 88–100% across all studies. Sensitivity of CK ranged from 47.5–83% across all studies. Specificity could not be calculated for the database studies as all the patients with NSTEMI were included.

Two studies (Wiens et al,^16^ and Ben Dor et al^10^) ultimately had a patient group diagnosed as an NSTEMI with a normal troponin and elevated CK. The Wiens et al data included a singular patient with a tenuous diagnosis of NSTEMI. The data from Ben Dor et al were unpublished and acquired through direct communication with the authors. This group represented 10.6% of their patient population; a greater proportion of their patients were troponin positive and CK negative (38%). Furthermore, in this study a large number of patients (24.6%) were diagnosed with NSTEMI in the face of both biomarkers being negative. The authors confirmed that no formal angiography, outcome, or echocardiography data were available for this cohort. As we have moved to a biomarker definition of NSTEMI, it is unclear whether the data from Ben Dor et al that were completely biomarker negative were misclassified or represent local practice patterns in diagnosis at the time.

Quality assessment showed that 12.5% and 25% of studies had high risk of bias and applicability concerns for patient selection (Appendix D).

## DISCUSSION

In this systematic review we found that none of the published results report that CK is useful for NSTEMI diagnosis when the troponin assay is negative. Two studies had evidence for such discordance where CK was elevated and troponin was negative. In one study the data were unpublished and in another represented just one patient. Overall, our systematic review showed that troponin is a superior biomarker with greater sensitivity and specificity. The overall low number of studies with complete data and the heterogeneity of the studies precluded a formal pooled meta-analysis of the data. Nevertheless, the data are in keeping with previous analysis of CK and troponin in ACS evaluation.^1^

As the cost of healthcare continues to rise, eliminating unnecessary testing from hospital departments will allow for better resource utilization. Depending on the institution and number of tests run yearly, thousands of dollars can be redirected to other patient care initiatives.^16^ Although we did not explicitly look at time to treatment, CK and cTn testing are generally both resulted within similar timeframes; therefore, eliminating CK should not result in any delays to diagnosis or treatment of NSTEMI.

## LIMITATIONS

The gold standard for NSTEMI diagnosis used in most papers was the WHO definition. This definition has evolved over the course of our study period and is the greatest limitation of our paper. The diagnosis of ACS (and NSTEMI) has evolved from requiring two of three of the following 1) clinical history of chest discomfort of >30 minutes duration, 2) evolution of typical ECG changes, and 3) rise and fall of serum enzymes (currently CK and its isoenzyme CK-MB),^15^ to our current diagnostic model of elevated biomarkers (cTn) with appropriate clinical context; ECG findings may be present but are not required.^7^ Many of the studies also used local criteria or the discharge diagnosis from their cardiology department as their reference standard for diagnosing NSTEMI. Finally, the diversity of the settings does not lend itself to a direct comparison or meta-analysis. Our review included chest pain patients on inpatient units, rural EDs, academic centers, and patients who were hospitalized for chest pain workup.

## CONCLUSION

Troponin (cTn) has become the mainstay of biomarker testing in NSTEMI diagnosis. This systematic review was able to identify one patient in published data, and a subset of unpublished data from one study with discordant biomarkers (CK positive when cTn was negative) for NSTEMI diagnosis. In the same studies the sensitivity of cTn surpassed CK. As expected, we found troponin far superior to creatine kinase with excellent sensitivity and specificity. The continued use of CK for NSTEMI diagnosis is no longer recommended.

## Supplementary Information



## Figures and Tables

**Table 1:** Characteristics of included studies.

Author	Study period	Study design	Setting	Total number of patients in study and total with diagnosis of NSTEMI	Discordant data	Sensitivity of troponin (cTn) (peak)	Sensitivity of CK (peak)
Apple et al, 1997^11^	1996–1996	Prospective	United States, Inpatient, NSTEMI database	48, 31 NSTEMI	No	100%	54%
Ben-Dor et al, 2006^9^	2002	Prospective	Israel, Inpatient, NSTEMI database	629, 629 NSTEMI	Yes, 10.6% (+ CK,−cTn)	91.3%	47.5%
Ishihara et al, 2017^10^	2012–2014	Retrospective	Japan, Inpatient, NSTEMI database	1,021, 1,021 NSTEMI	No	100%	55%
Ferguson et al. 2002^12^	2002	Prospective	Scotland, Inpatient admitted from ED	80, 13 NSTEMI	No	100% (0.75–1.0)	69% (0.39–0.91)
Graven et al. 2001^13^	1998–1999	Prospective	Norway, Inpatient admitted from ED	442, 130 NSTEMI	No	100% (0.97–1.0)	50% (0.44–.58)
Hindle et al, 2005^14^	2001–2002	Retrospective	Canada, ED	235, 11 NSTEMI	No	90% (0.55–1.0)	83% (0.78–0.88)
Tucker et al, 1997^15^	1997	Prospective	United States, Inpatient admitted from ED	177, 27 NSTEMI	No	89% (0.71–0.98)	81% (0.62–0.94)
Wiens et al, 2019^16^	2017	Retrospective	Canada, ED	9,951, Total NSTEMI not reported	Yes, 0.012% (+ CK,−cTn)	Data not available	Data not available

*CK*, creatine kinase; *ED*, emergency department; *NSTEMI*, non-ST-elevated myocardial infarction; *cTn*, troponin I, troponin T.
