# Supplementary material for: Role of Creatine Kinase in the Troponin Era: A Systematic Review
Source: West J Emerg Med. 2021 Oct 27;22(6):1291–4. doi: 10.5811/westjem.2020.11.47709 (PMC8597688; doi:10.5811/westjem.2020.11.47709)

**Appendix A.** Search strategy.

Search Terms:

myocardial ischemia/ or acute coronary syndrome/ or coronary artery disease/ or coronary occlusion/ or coronary stenosis/ or coronary thrombosis/ or myocardial infarction/ or anterior wall myocardial infarction/ or inferior wall myocardial infarction/ or non-ST elevated myocardial infarction/
(myocardial adj2 infarct*).ti,ab.
(heart adj2 attack*).ti,ab.
Creatine kinase/
(phosphokinase adj2 creatine*).ti,ab.
(creatine adj2 phosphotransferase*).ti,ab.
Troponin C/ or Troponin/ or Troponin T/ or Troponin I/
troponin.ti,ab.
1 or 2 or 3
4 or 5 or 6
7 or 8
9 and 10 and 11
limit 12 to (English language and humans and yr = "1995 -Current")

**Appendix B.** Article selection**.**


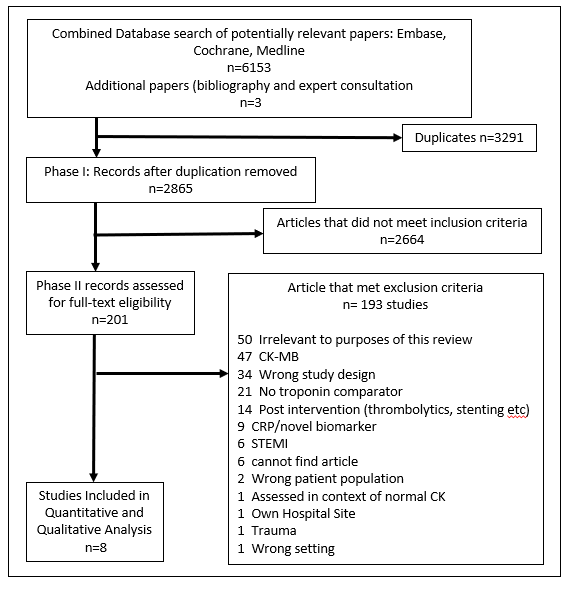


**Appendix C.** Sensitivity tables of selected studies**.**

Troponin (cTn)


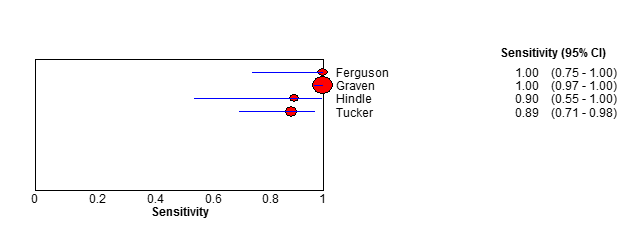


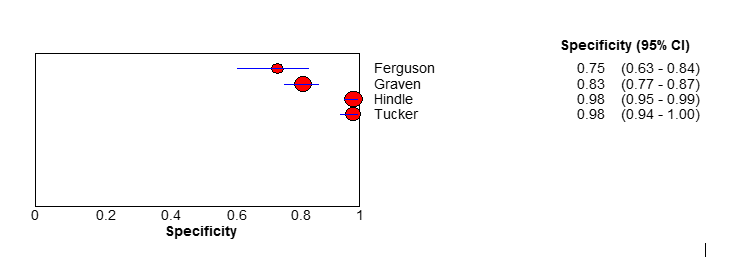


Creatine Kinase (CK)


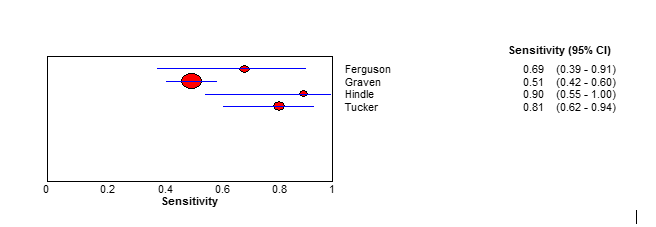


**r
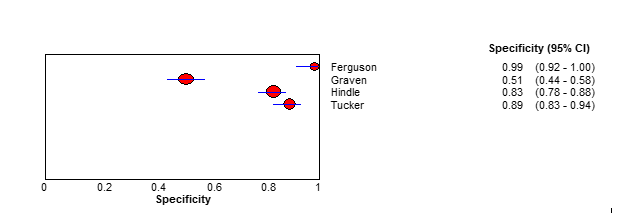
**

*CI*, confidence interval.

| **Appendix D.** Risk of bias assessment using QUADAS- 2. |  |  |  |  |  |  |  |  |
| --- | --- | --- | --- | --- | --- | --- | --- | --- |
| QUADAS2 tool was used to evaluate the risk of bias and applicability in the domains of patient selection, index test, reference standard, and flow and timing. |  |  |  |  |  |  |  |  |

| **Study** | **Risk of Bias** |  |  |  |  | **Applicability** |  |  |
| --- | --- | --- | --- | --- | --- | --- | --- | --- |
|  | Patient Selection |  | Index Test | Reference Standard | Flow and timing | Patient Selection | Index Test | Reference Standard |
| Apple | Low |  | Low | Unclear | Low | High | Low | Low |
| Ben Dor | Low |  | Low | Low | Unclear | Low | High | Low |
| Ferguson | Low |  | Low | Low | Low | Low | Low | Low |
| Graven | Low |  | Low | Low | Low | Unclear | Low | Low |
| Hindle | High |  | Low | Unclear | High | High | Unclear | Unclear |
| Ishihara | Low |  | Low | Low | Low | Low | Low | Low |
| Tucker | Low |  | Low | Low | Low | Low | Low | Low |
| Wiens | Low |  | Low | Low | Low | Low | Low | Low |

Proportion of studies with low, high, or. unclear risk of bias.


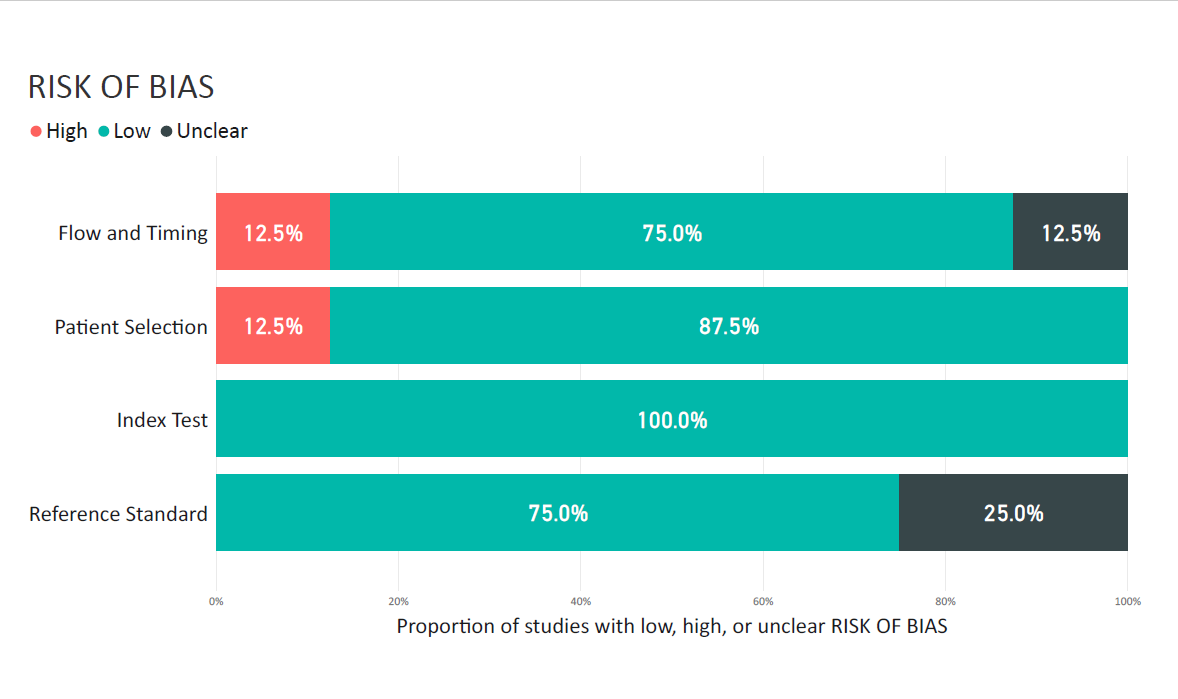


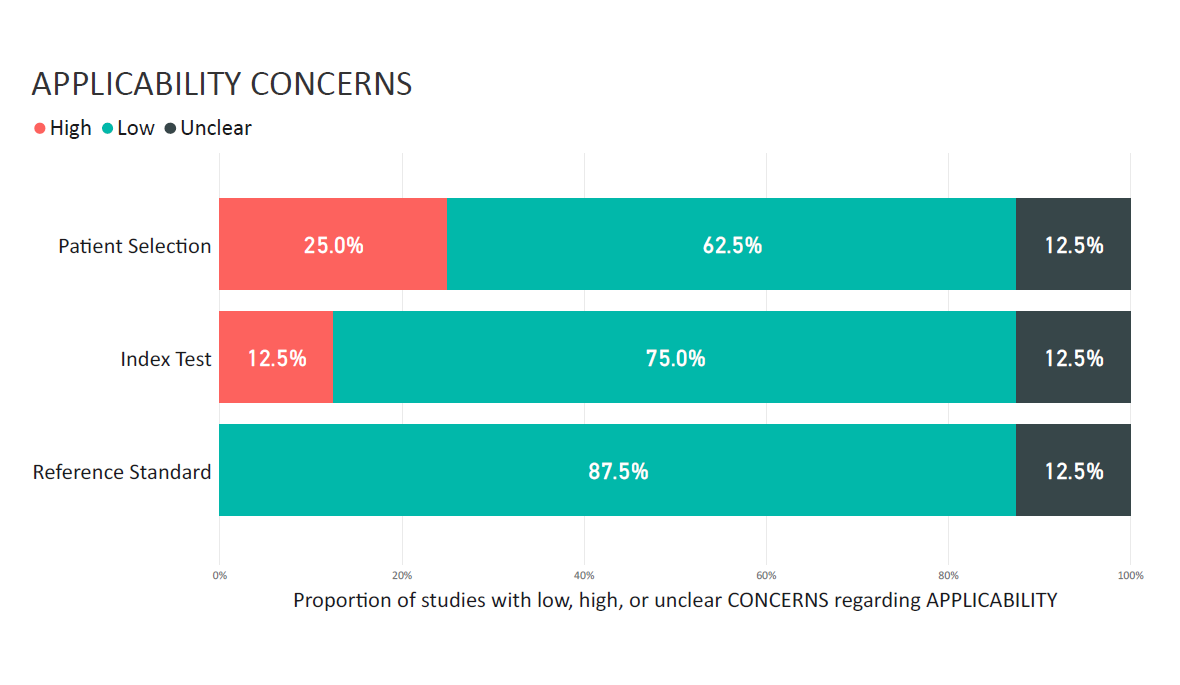

Supplement: Supplementary file 1 [file wjem-22-1291-s001.docx]
